# Supplementary material for: Direct detection of a single photon by humans
Source: Nat Commun. 2016 Jul 19;7:12172. doi: 10.1038/ncomms12172 (PMC4960318; doi:10.1038/ncomms12172)
Supplement: Supplementary Information — Supplementary Figures 1-6, Supplementary Tables 1-3, Supplementary Note 1-3 and Supplementary References [file ncomms12172-s1.pdf]

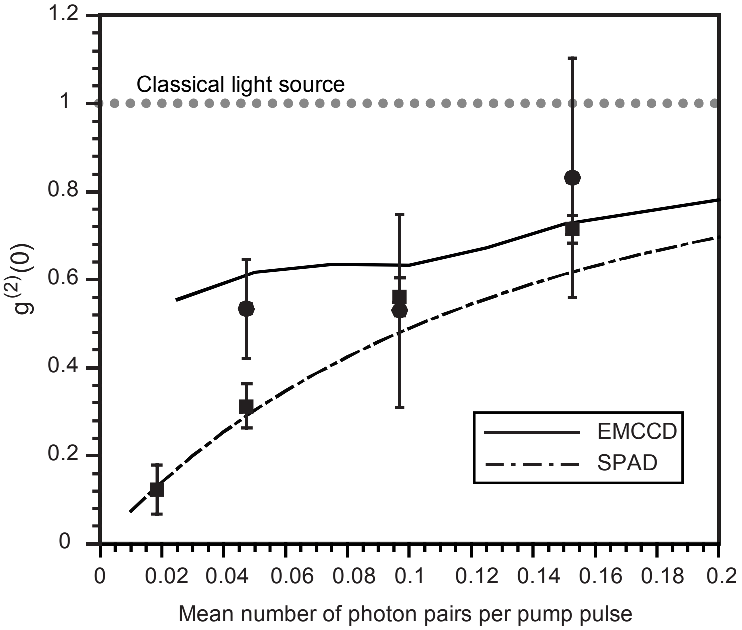

Supplementary Figure 1. Characterization of the single-photon quantum light source based on spontaneous parametric down-conversion (SPDC).

Experimentally measured $g^{(2)}(0)$ values of the SPDC source using an SPAD (squares) and an EMCCD (circles) as a detector in the idler arm as a function of SPDC pump power (mean photon pairs per pulse). Lines: numerical simulations taking into account the parameters and theory of operation of the SPAD (dashed) and the EMCCD (solid) (see Methods for details). Gray dotted line shows performance of classical light source with $g^{(2)}(0)=1$, i.e. Poisson distributed. Error bars denote s.e.m.

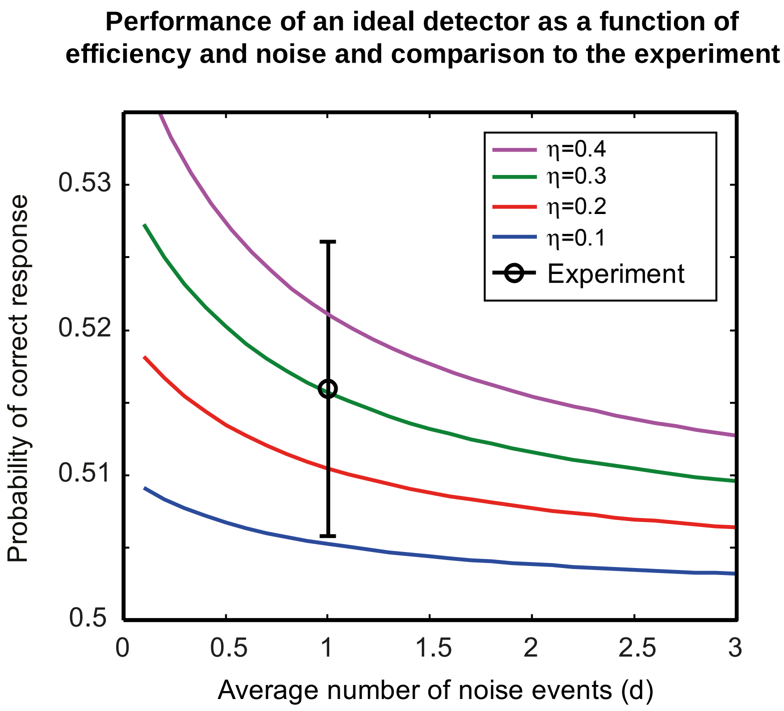

Supplementary Figure 2. Performance of an ideal detector in two alternative force choice (2AFC).

Simulations of the performance of an ideal detector in 2AFC trials based on signal detection theory (SDT). Performance decreases as a function of average number of noise events per integration time. Baseline at 0.5 corresponds to performance expected from random guessing. Different colors denote simulations with different parameters of efficiency (η). Range for d and η based on data on physiological measurements (Supplementary Table 1). Experimental data is shown as black circle with error bar at the estimated level of noise.

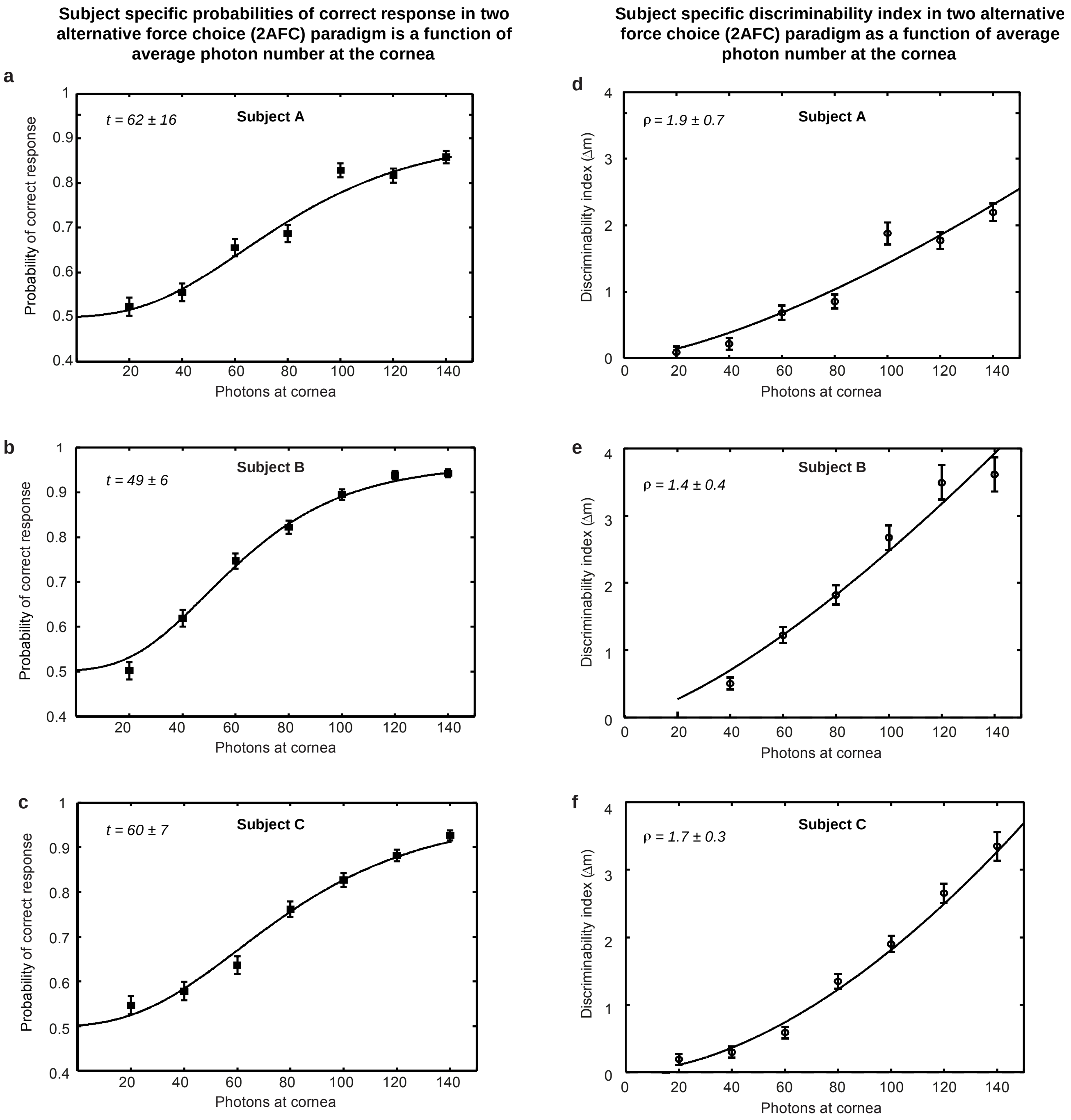

Supplementary Figure 3. Subject specific probabilities for giving a correct answer in the high photon range.

(a) - (c) The probability of correct response for the high photon range (20-140) using classical Poisson light for combined confidence ratings subjects A, B and C respectively. Solid lines are fits used to extract the sensitivity threshold, defined as number of photons required to reach a performance level equal to the half of the maximum value, indicated as a number in top left corner. Baseline at 0.5 corresponds to random guessing performance. **(d) - (f)** Corresponding discriminability index (Δm) (Supplementary Note 2). Solid lines show power fits to the data ~ x^{ρ}. Value of the fit parameter is shown on the plot.

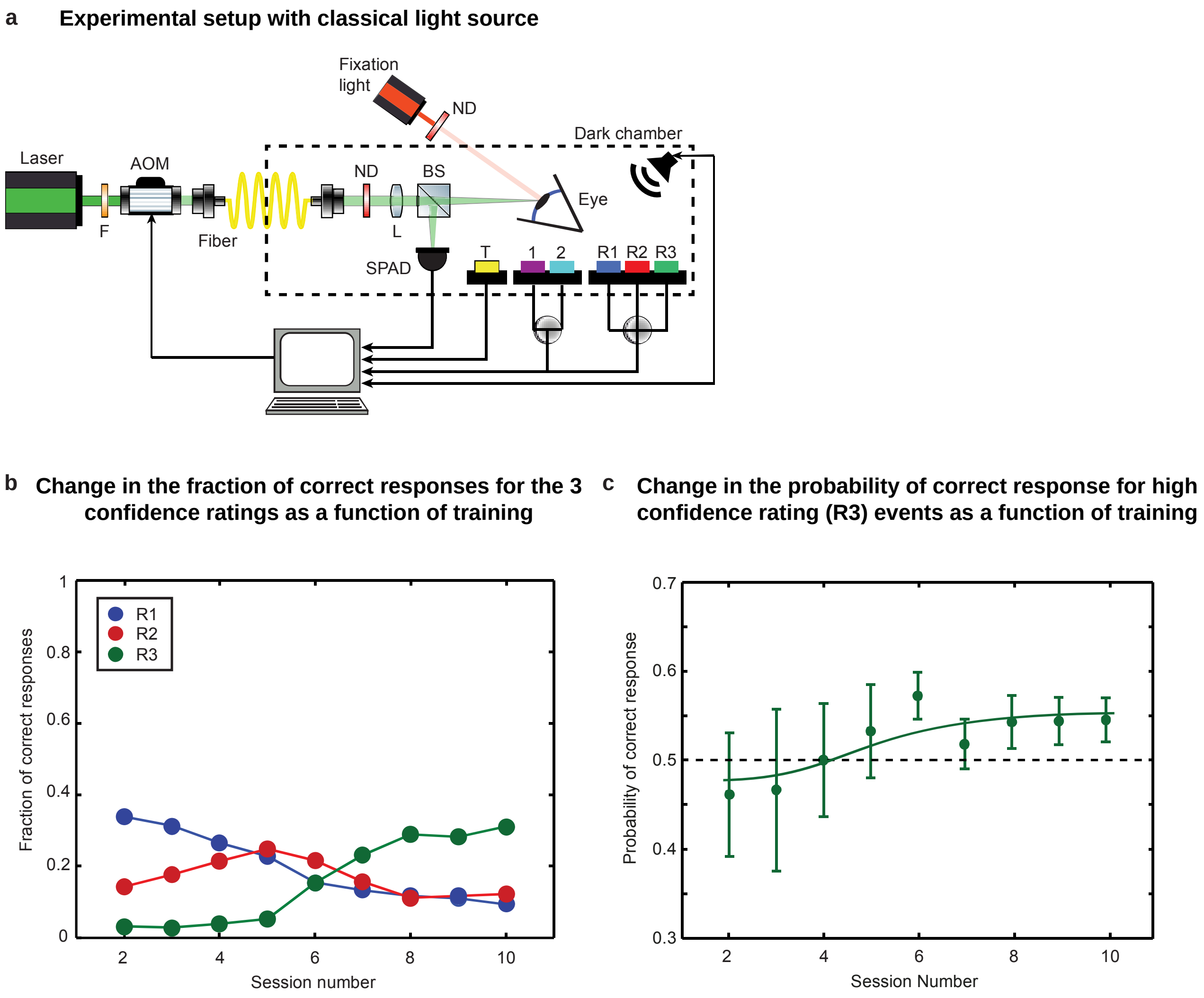

Supplementary Figure 4. Experimental setup with classical light source and improvement of performance with training at low (0-15) photon numbers.

(a) Schematic of the experimental setup with classical light source. Light stimuli from a ~500 nm laser source, attenuated by an acousto-optic modulator (AOM), are triggered (T) by subjects and coupled into a single-mode fiber entering the dark chamber. The light is directed and focused onto the pupil (Maxwellian view) at an angle of 23° temporal to a highly attenuated red fixation light presented at the fovea. F, bandpass filter, ND, neutral density filter, L, lens, BS, 50/50 beam-splitter, SPAD, single-photon avalanche detector. **(b)** Fraction of correct responses for the three confidence ratings (R1, R2, R3) as a function of training sessions for photons in the photon range 0-15 photons at the cornea. Data was pooled across subjects. Fraction of high confidence (R3) ratings increases with training. **(c)** The probability of correct response for combined subjects and for R3 answers shows improvement with session number. Baseline at 0.5 corresponds to random guessing level. Error bars denote s.e.m.

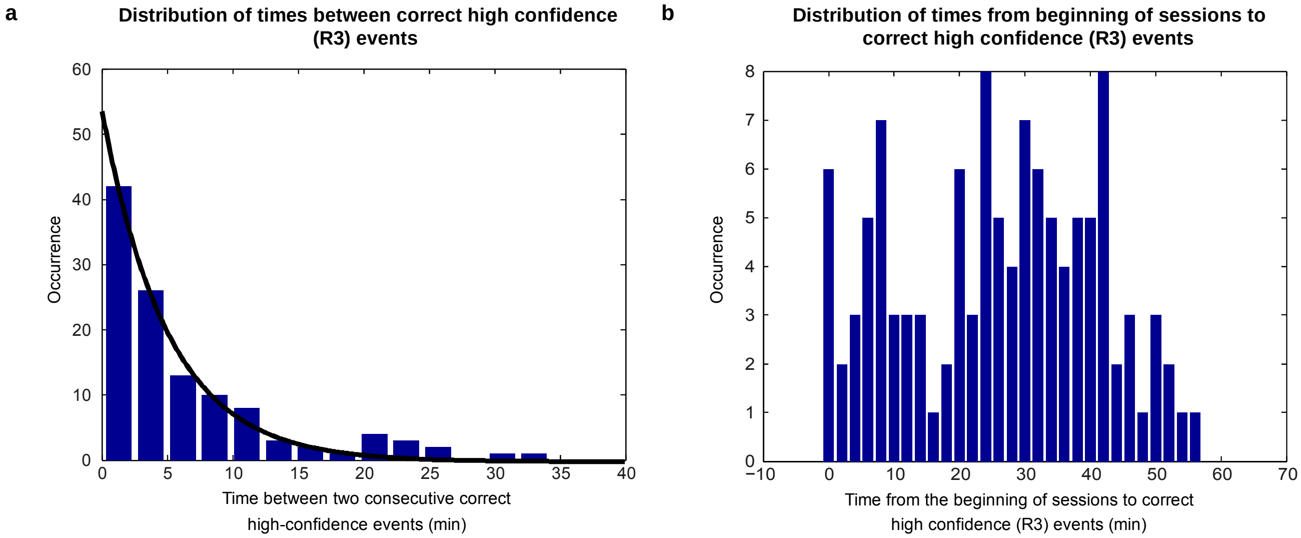

Supplementary Figure 5. Timing analysis of correct high confidence (R3) events in SPDC single-photon trials.

(a) Distribution of times between the correct high confidence (R3) events in all sessions and fit to the exponential function (solid line). Data from all subjects. **(b)** Distribution of the times of correct high confidence events with respect to the beginning of the sessions for combined subjects and sessions (at time point 0). The distribution is not statistically different from uniform on 0-60 min ($p = 0.07$).

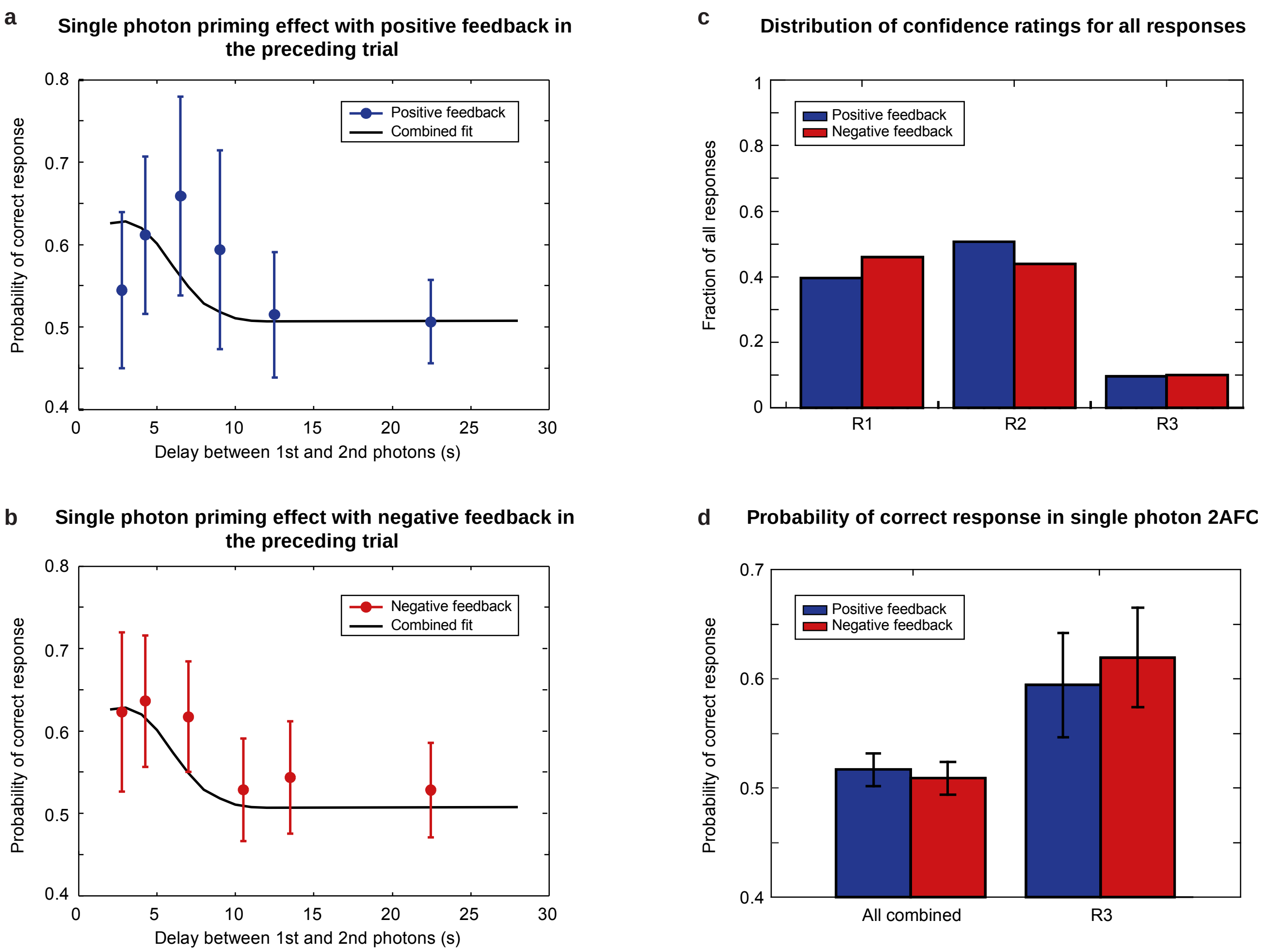

Supplementary Figure 6. Effect of positive and negative feedback in single-photon experiments.

(a), (b) Probability of correct response averaged across subjects and ratings for single-photon trials for which subjects received positive **(a)** and negative **(b)** feedback as a function of time to the preceding single photon. Solid line is fit of the combined data (Fig. 2c). **(c)**, **(d)** Distribution of ratings and probability of correct response in single-photon post-selected trials. Only those trials whose preceding trial was blank were chosen. Blue and red bars are plotted based on the feedback received in the previous blank trial. Lack of significant difference between data for positive and negative feedback suggest that higher order unspecific (cortical) effect are unlikely to mediate the observed single-photon induced priming effect.

Source	Wavelength	Transmission from cornea to retina	Fraction absorbed by retina	Efficiency of producing output signal	Overall
Ambach, W. et al. Spectral transmission of the optical media of the human eye with respect to keratitis and cataract formation. Documenta ophthalmologica. Advances in ophthalmology 88, 165-173 (1994).	450 - 700 nm	> 80%			
Barlow, H. B. Retinal and central factors in human vision limited by noise., in Vertebrate photoreception. ed H.B. Barlow (Academic Press, 1977).	500 nm	50 - 75%	50 - 55%	60 - 100%	11- 33%
Boettner, E. A. & Wolter, J. R. Transmission of the Ocular Media. Invest Ophthalmol Vis Sci 1, 776-783 (1962).	500 nm	max 83.5%			
Donner, K. Noise and the absolute thresholds of cone and rod vision. Vision Res 32, 853-866 (1992).	500 nm	50 - 75%	80 - 93%	60 - 70%	17- 39%
Hallett, P. E. Quantum efficiency of dark-adapted human vision. J Opt Soc Am A 4, 2330-2335 (1987).	507 nm				12- 30% (*)
Norren, D. V. & Vos, J. J. Spectral transmission of the human ocular media. Vision Res 14, 1237-1244 (1974).	500 nm	75%			
Rieke, F. & Baylor, D. A. Single-photon detection by rod cells of the retina. Reviews of Modern Physics 70, 1027-1036 (1998).	~ 500 nm		~ 50%	60 - 67%	
Xu, J., Pokorny, J. & Smith, V. C. Optical density of the human lens. J Opt Soc Am A Opt Image Sci Vis 14, 953-960 (1997)	430 - 740 nm	95.3% (only lens)			

*This value was estimated based on psychophysics studies as theoretically possible maximal range

Supplementary Table 1. Summary of the efficiency estimates in the visual systems based on physiological measurements.

The overall efficiency is the combined effect of transmission through the ocular media from cornea to the retina (column 3), efficiency of absorption (column 4) and efficiency of producing the output signal (column 5). The end column shows the product of the preceding three columns.

Camera Parameter	Value
Read-Out Speed	1 MHz
Vertical Shift Speed	0.3 μs
Vertical Shift Amplitude	+2 V
Pre-Gain	3
Gain	1000
Exposure time	100 μs
Temperature (water-cooled)	-80 °C

Supplementary Table 2. Summary of EMCCD camera settings used when in (single-) photon counting mode.

N	Parameter	Description	95% confidence range from posterior distribution
1	t_{lag}	Lag time between an isomerization and the beginning of the increase in gain	0 - 1 s
2	T_{max}	Time at which the gain reaches its maximal value	2.7 - 4 s
3	α	Parameter defining the profile of the rising part of the gain function	0.1 - 1
4	β	Parameter defining the profile of the decreasing part of the gain function	4.6 - 6
5	A	Maximal amplitude of the gain	1 - 6.2
6	*d*	Average number of photon-like noise events per area per integration time	0.2 - 1
7	*P*	Average number of photon events due to single photon incident on the eye	0.2 - 0.48
8	C2	Desicion criterion separating low and medium confidence ratings	0.9 - 1.2
9	C3	Desicion criterion separating medium and high confidence ratings	3.2 - 4

Supplementary Table 3. Parameters and their inferred values for the model of the photon-induced temporal modulation of single-photon light perception.

Supplementary Note 1. Performance of an ideal detector in 2AFC trials

Signal detection theory (SDT) provides a framework for analyzing detection in psychophysics tasks. In SDT the sensory process is assumed to have a continuous output based on a distribution of noise or noise combined with a signal. In a visual process in its simplest assumption, noise and noise-plus-signal distributions are appropriately described as Poisson. If there are no other sources of noise except for irreducible Poisson fluctuations, the probability of detecting n noise events is described by,

$$p_d(n) = \frac{d^n}{n!} e^{-d} \quad (1)$$

where d is the mean amount of noise events per integration interval. The probability of detecting k events within the interval containing noise and signal is:

$$p_s(k) = \frac{S^k}{k!} e^{-S} \quad (2)$$

where S is the average number of signal plus noise events, and $S=d+\eta E$ where E is the average number of photons at the cornea, η is the quantum efficiency of the system,

SDT assumes that the detector's decision in a 2AFC task is determined by the likelihood ratio [1]:

$$L(x_1, x_2) = \frac{f(x_1, x_2 \,|\, <sn>)}{f(x_1, x_2 \,|\, <ns>)} \quad (3)$$

where $f(x_1, x_2 \,|\, <sn>)$ and $f(x_1, x_2 \,|\, <ns>)$ are two distributions for the alternative cases where the signal was presented in the first or in the second interval, respectively,

and x_1 and x_2 are respectively the number of events in the first and second intervals. Using Poisson likelihood, the log likelihood is simplified to:

$$\ln L(x_1, x_2) = (x_1 - x_2)\ln\frac{S}{d} \tag{4}$$

We define an ideal detector as an idealistic observer, whose performance is only limited by the presence of noise and the quantum efficiency. In making a decision for a 2AFC trial, an ideal detector will maximize the likelihood and therefore pick the interval based on the difference x_1 - x_2 [1]. Assuming the ideal observer guesses randomly when $x_1 = x_2$, the probability of correct response is:

$$P(C) = \frac{1}{2}p_{diff}(0) + \sum_{i>0} p_{diff}(i) \tag{5}$$

where $p_{diff}(i)$ is the distribution of the difference S - d.

In the ideal case, the probability of correct response depends only on the two parameters d and η. Assuming an integration time of 100 ms and using known rates of dark isomerizations [2], this equates to ~ 1 photon per trial interval in our experiments (see also Supplementary Note 3). We have plotted the probability of correct response for the ideal detector as a function of the average number of photon events per integration time (d) for different values of quantum efficiency (η) (Supplementary Fig. 2).

The model of an ideal detector also allows for studying how bias in the subject's responses towards the first or second interval might influence their overall performance. In the ideal case and in the limit of an infinite amount of experimental

trials, the presence of a bias can only decrease the overall performance and cannot explain any increase [1]. Our data indicated that on average our subjects tended to choose the second interval over the first 16% more frequently, which would result in an insignificant degradation of performance.

In reality, the finite number of post-selected trials (n=2420) might have resulted, due to statistical variation, in more presentations in, e.g., the second interval. The likelihood that elevated performance results from such an effect can be evaluated using Fisher's statistical test, given the contingency matrix of our data. This analysis results in p=0.05 for all responses and p=0.001 for high confidence responses (R3). Therefore, subjects' bias cannot account for the observed performance of correctly detecting single photons.

Supplementary Note 2. Estimating the discriminability index Δm from experimental data

Signal detection theory is widely used to extract parameters of underlying noise and signal plus noise distributions from experimental data. The distance between the means of the signal plus noise and the noise distributions in standard deviation units is called d' (d-prime), and can be calculated from the data [1]. In the visual system, detection is described by Poisson distributions, which have different variances for different means. Therefore we have chosen to calculate the index of discriminability Δm, which like d' is a measure of the separation between the noise and the signal-plus-noise distributions, but can be calculated for distributions with different variances:

$$\Delta m = \frac{S-d}{\sigma_N}, \tag{6}$$

where S is the mean of the signal plus noise distribution, d is the mean noise, σ_N is standard deviation of the noise distribution, with $\sigma_N = \sqrt{d}$.

The probability of correct response is related to S and d, and therefore Δm via equation (5) (Supplementary Note 1). We used it to infer the Δm value from the known probability of correct response in our 2AFC trials.

Supplementary Note 3 Model for the photon-induced temporal modulation of single-photon light perception

Single-photon perception priming (Fig. 2c,d) is unlikely to represent an unspecific higher brain cognitive effect (see Methods), and we speculate that it rather represents a part of the retinal processing circuitry. We aimed to develop a mathematical model of the visual system that would allow us to study how the probability of giving the correct answer and, more generally, the response of the subject, depends on the parameters of the system. All parameters of the model are summarized in Supplementary Table 3.

We assume that each photon-like event, irrespective of stemming from light or dark noise, induces a transient modulation of the gain. In order to account for a possible lag period and for potential different profiles to the rise and fall of the gain, we introduced the following general function to describe how the gain changes in time following an event at t=0:

$$G(t) = \begin{cases} 0 & t < t_{lag} \\ (t - t_{lag})^{\alpha} \exp\left\{-\alpha \dfrac{(t - t_{lag})}{T_{\max}}\right\} & t_{lag} < t < t_{lag} + T_{\max} \\ (t - t_{lag})^{\beta} \exp\left\{-\beta \dfrac{(t - t_{lag})}{T_{\max}}\right\} & t > t_{lag} + T_{\max} \end{cases} \quad (7)$$

where t_{lag} is the lag time, T_{max} is the time at which the gain reaches its maximum, and α and β determine the profiles of the rising and declining regions of the curve, respectively (Fig. 3c).

This time-dependent gain has an additional dependence upon the random number of noise events in the time intervals preceding the first and second experimental intervals. We assumed that all those events average to form a continuous and stationary background gain. Using these simplifications, we calculated numerically the probability of correct response and the relative change in first interval responses.

In order to calculate the probability of correct response or the fraction of first interval responses, we simulated 10^4 – 10^5 trials and averaged outcomes across them. In each trial, random numbers were drawn to determine whether a photon was present before or during the trial. This was done sequentially in time: the presence of a photon before the trial affects the gain of both intervals.

For a given gain, the average signal due to photon and photon-like noise events at is given by:

$$E = G(t) \cdot (P + d) \quad (8)$$

and

$$E = G(t) \cdot d \text{, otherwise.} \quad (9)$$

where $G(t)$ is the time-dependent element of the gain of the system, d is the average number of photon-like noise events during an interval, and P is the average number of light-induced photon events.

We assumed that the amplified signals from the two intervals are compared and that the one, probabilistically, containing the higher signal is chosen. In the case of no events being detected in either interval or of the same number of events being detected in both intervals, we assumed the subject to choose randomly between the first and second intervals.

We aimed to find a joint parameter set capable of simultaneously fitting the data in both Fig. 2c and Fig. 2d. In order to achieve this we used an optimization strategy based on Bayesian inference. 90% confidence intervals calculated from posterior distributions are presented in the Supplementary Table 3, with parameters 1 – 5 defining the shape of the gain function. Noise events were inferred to be in the range 0.1 – 0.6 mean photon-like noise events per integration interval per spatial area. This number is in agreement with known estimates for the number of dark isomerizations in rod cells [2].

Parameter 7 might be interpreted as the efficiency of the system, yielding the probability of the retinal detection of an event when a single photon impinges upon the cornea. Previous psychophysics measurements place the quantum efficiency of an eye between 0.03 and 0.23, while direct estimates based on losses within the eye range from 0.1 to 0.4 (Supplementary Table 1). Interestingly, our estimate based on the psychophysics data that takes into account the effect of temporal gain enhancement is

more consistent with the one based on transmission measurements. It is therefore plausible that under the lowest light conditions used in the study, the efficiency of vision reaches its potential maximum set by the physical properties of the ocular medium.

In addition, we asked whether the presence of two decision criteria applied at the level of amplified signals could account for the observed higher performance for high confidence events (Fig. 2a). We introduced criteria *C2* and *C3*, which separate the possible difference between signals equation (8) and equation (9) into 3 ranges (Fig. 3a,b). When the difference was smaller than *C2* the event was counted as a rating 1 event, between *C2* and *C3* as a rating 2 event and higher than *C3* as a rating 3 event.

The probability of correct response and fraction of all responses were calculated as following:

$$P_{Ri}(C) = \int_{t=0}^{\infty} p_{Ri}(t) \cdot T(t)dt \tag{10}$$

$$F(R_i) = \int_{t=0}^{\infty} f_{R_i}(t) \cdot T(t)dt \tag{11}$$

Where $P_{Ri}(C)$ is the probability of correct response for rating R_i events, and $F(R_i)$ is fraction of R_i events. Here $p_{Ri}(t)$ is the probability of giving the correct response for R_i events as a function of time to the previous single-photon event, $f_{R_i}(t)$ is the fraction of rating R_i events as a function of time to the previous single-photon event, $T(t)$ is the distribution of time to the previous single-photon events extracted from the data.

In order to find coefficients $C2$ and $C3$ we kept the best parameter fit identified for the combined probability of correct response and relative change in interval 1 answers temporal functions (Fig. 3a,b) and varied $C2$ and $C3$ until we found the best combined fit for the probability of correct response as a function of delay for rating 3 events (Fig. 2c inset), total probability of correct response (Fig. 3d) and the fraction of all responses assigned to each rating in the SPDC experiments (Fig. 3f).

Effect of feedback received on the performance of subjects

Our model suggests that temporal enhancement of the gain occurs on a time scale of several seconds following a photon detection. This makes it unlikely that this effect occurs at the level of rod cells, whose response is very fast [3]. We speculated that this might be a processing mechanism in the retina (see main text).

In order to exclude the possibility that this temporal enhancement comes from higher brain cognitive effects, we plotted the probability of the correct response as a function of time to the previous photon and feedback received in the previous interval. Supplementary Fig. 6a and 6b show that enhancement is not correlated with the feedback received in the previous trial.

Next, we used trials in the SPDC experiments in which by random chance no photons were presented to the subject but nonetheless the feedback was received based on the interval in which the photon would have been. We found that the distribution of ratings and the probability of correct response on trials following such events do not depend on the type of feedback received by the subject (Supplementary Fig. 6c,d), suggesting that the observed effect was not due to increased attention on the part of the subject.

Supplementary References

1 Green, D. M. & Swets, J. A. *Signal Detection Theory and Psychophysics*. reprint edn, (Peninsula Publishers, 1989).

2 Baylor, D. A., Nunn, B. J. & Schnapf, J. L. The photocurrent, noise and spectral sensitivity of rods of the monkey Macaca fascicularis. *J Physiol* **357**, 575-607 (1984).

3 Field, G. D., Sampath, A. P. & Rieke, F. Retinal processing near absolute threshold: from behavior to mechanism. *Annu Rev Physiol* **67**, 491-514 (2005).
